# How I do it? Uniportal full endoscopic contralateral approach for lumbar foraminal stenosis with double crush syndrome

**DOI:** 10.1007/s00701-019-04157-z

**Published:** 2019-12-10

**Authors:** Pang Hung Wu, Hyeun Sung Kim, Il-Tae Jang

**Affiliations:** 1 Department of Neurosurgery, Nanoori Gangnam Hospital, 731, Eonju-ro, Gangnam-gu, Seoul, Republic of South Korea; 2 JurongHealth Campus, Orthopaedic Surgery, National University Health Systems, Singapore, Singapore

**Keywords:** Uniportal endoscopy, Contralateral approach, Foraminal stenosis, Minimally invasive spine, Endoscopic spine, Spinal stenosis, Degenerative spine, Spine decompression

## Abstract

**Background:**

Evolution of endoscopic surgery provides equivalent results to open surgery with advantages of minimal invasive surgery. The literature on technique Uniportal Full endoscopic contralateral approach is scarce.

**Methods:**

The endoscopic contralateral approach technique applies for patients presenting with double crush syndrome with foraminal and extraforminal stenosis. The key steps focus on contralateral ventral overriding superior articular process decompression, foraminal and extraforaminal discectomy, and lateral vertebral syndesmophyte decompression leading to enlargement of the contralateral foramen and extraforamen size.

**Conclusion:**

The Uniportal Full endoscopic contralateral approach is a good alternative to open surgery or minimally invasive microscopic surgery through direct endoscopic visualization of the entire route of exiting nerve with no neural retraction allowing both lateral recess and foraminal and extraforaminal decompression all in one approach.

**Electronic supplementary material:**

The online version of this article (10.1007/s00701-019-04157-z) contains supplementary material, which is available to authorized users.

## Relevant surgical anatomy

**Fig. 1:**
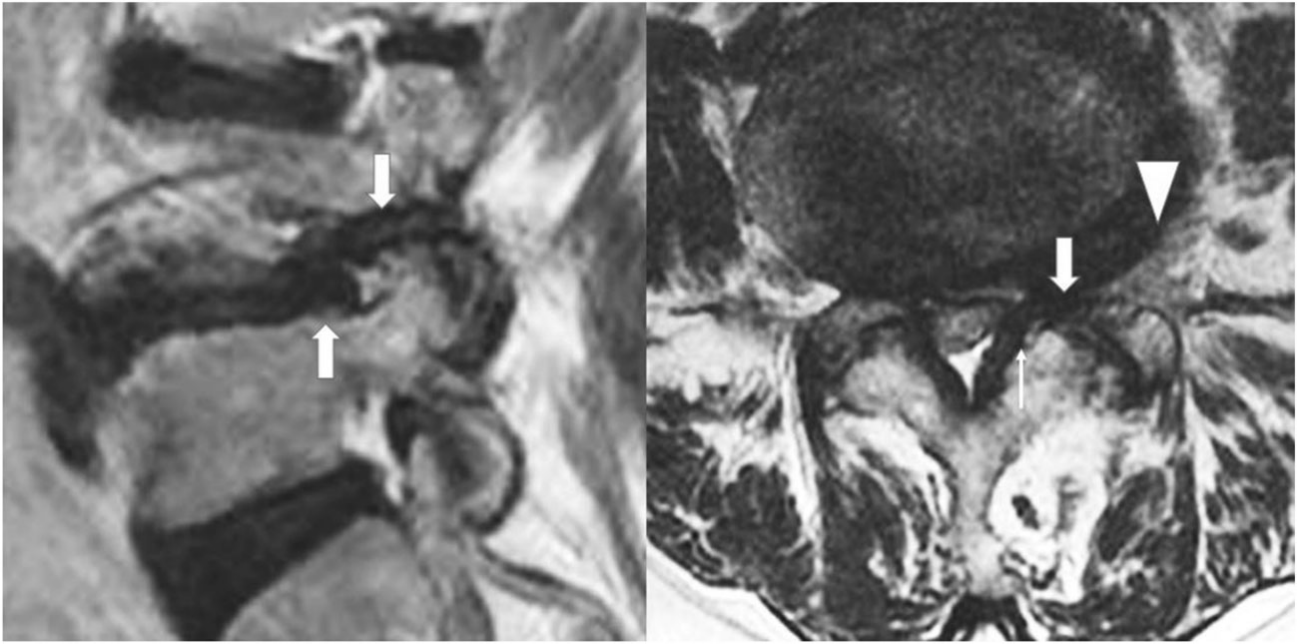
Double region of potential crush on a degenerative spine. In the lateral recess region on axial view (narrow arrow, there is thickening of the ligamentum flavum with paracentral disk protrusion and annular tear). In the foraminal region (broad arrow) in both sagittal and axial cuts showing overriding of superior articular facet, thickened and buckled ligamentum flavum and foraminal disk protrusion leading to foraminal stenosis. In the extra foraminal region, a disk protrusion can compress on the exiting nerve root running along the extraforaminal disk (broad arrow head)

In lumbar spine spinal stenosis, we tend to have multiple points [2, 3] of (a) compression with paramedian hypertrophic ligamentum flavum and paramedian disk protrusion leading to lateral recess stenosis; (b) foraminal compression with foraminal disk protrusion, syndesmophytes, and facet hypertrophy decreasing the anteroposterior diameter and/or overriding superior articular facet decreasing foraminal height with pedicle impingement; and (c) extraforaminal disk compression (Fig. 1). Current open, microscopic, and biportal endoscopic techniques tend to require 2 separate approaches to decompress lateral recess, foraminal, and extraforaminal compression of the nerve root. Asymmetrical lumbar stenosis with correlated clinical and radiological findings is suitable for uniportal full endoscopic contralateral approach decompression with the benefit of one approach addressing multiple points of compression and being minimally invasive especially in the elderly [5–7].

Ligamentum flavum is attached to the ventral lower half of the cephalad lamina, ventral-dorsal superior edge of the caudal lamina, and the ventral-superior articular process [9]. Complete contralateral ligamentum flavum resection and bony and disk decompression with free flowing neural element is key to success of the surgery.

## Description of the technique

### Clinical and radiological investigation to rule out instability contralateral approach corridor

Clinical findings with concordant imaging are key in patient selection. Anteroposterior, lateral, lumbar flexion, and extension roentgenogram should be obtained to rule out anteroposterior and lateral spinal instability [1, 8].

Careful analysis of CT and MRI is carried out in planning on the amount of foraminal decompression with consideration of the anteroposterior and superior–inferior diameter of foramen. Such planning avoids unnecessary endoscopic foraminal exploration (Fig. 1).

### Anesthesia and positioning

The patient is positioned prone on a Wilson frame on a radiolucent table. The procedure is done under epidural anesthesia with monitored sedation. The surgeon stands opposite to the side of lesion.

### Skin marking

**Fig. 2:**
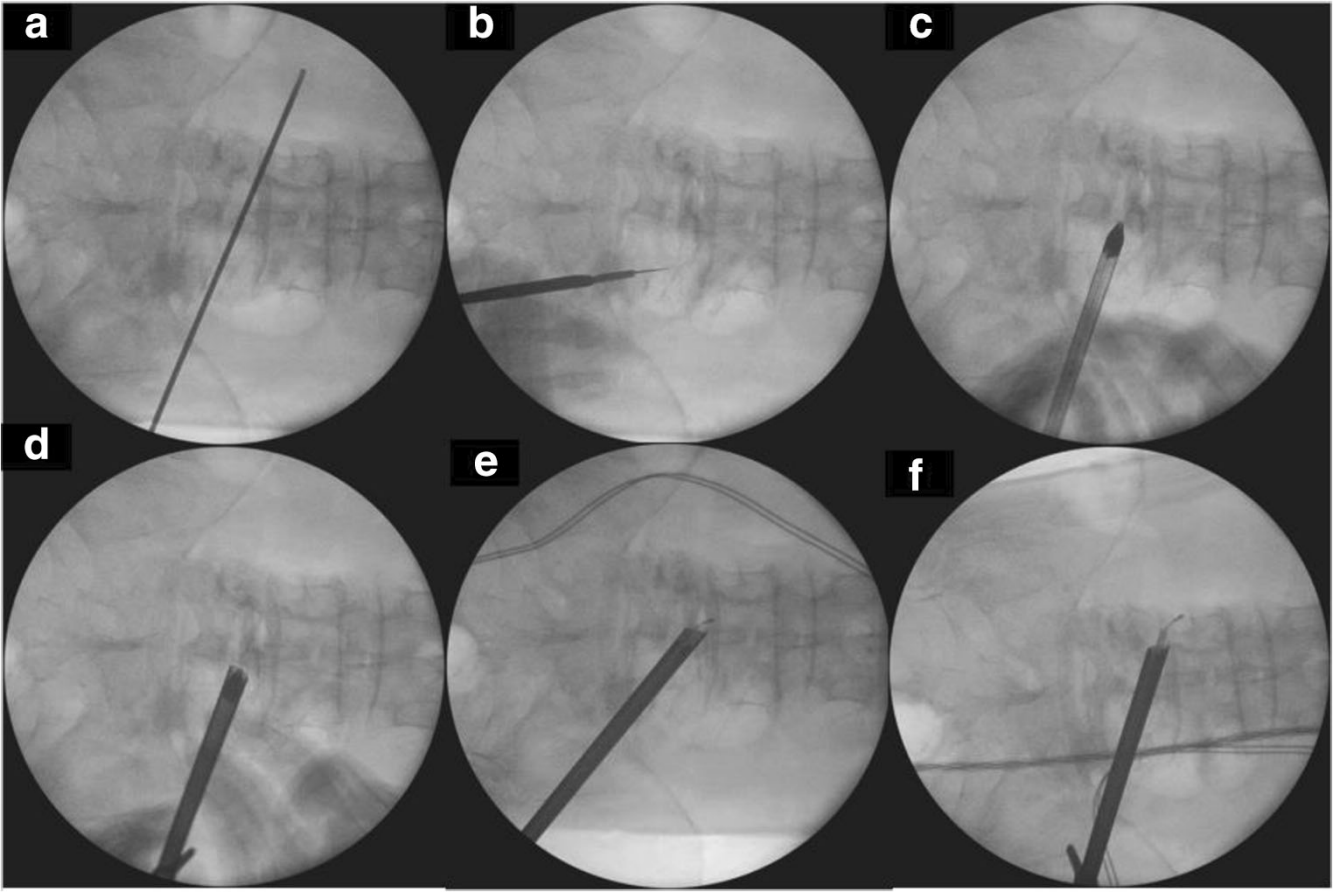
Marking on the intersection of the line pointing to the foramen with the line parallel to the medial pedicle (**a**, **b**). After skin incision, the dilator is docked directly on the spinolaminar junction with the docking of the working channel over the dilator (**c**, **d**). After initial sublaminar decompression over the top of flavum and dura in midline, endoscope traverse to contralateral side (**e**). Complete foraminal and extraforaminal decompression (**f**)

Skin incision is the intersection of extrapolation of the oblique line parallel to the contralateral foramen with the line directly over the lateral laminar window intersection with the facet joint of the ipsilateral side (Fig. 2a, b).

### Docking and Working Channel placement

Docking is done on the spinolaminar junction of the ipsilateral side with obturator with gradual dilation to open the bevel working channel (Fig. 2c, d) We use a 30°/7.3-mm-outer diameter/171-mm-length endoscope (Fig. 2e, f).

### Translaminar vs sublaminar approach

**Fig. 3:**
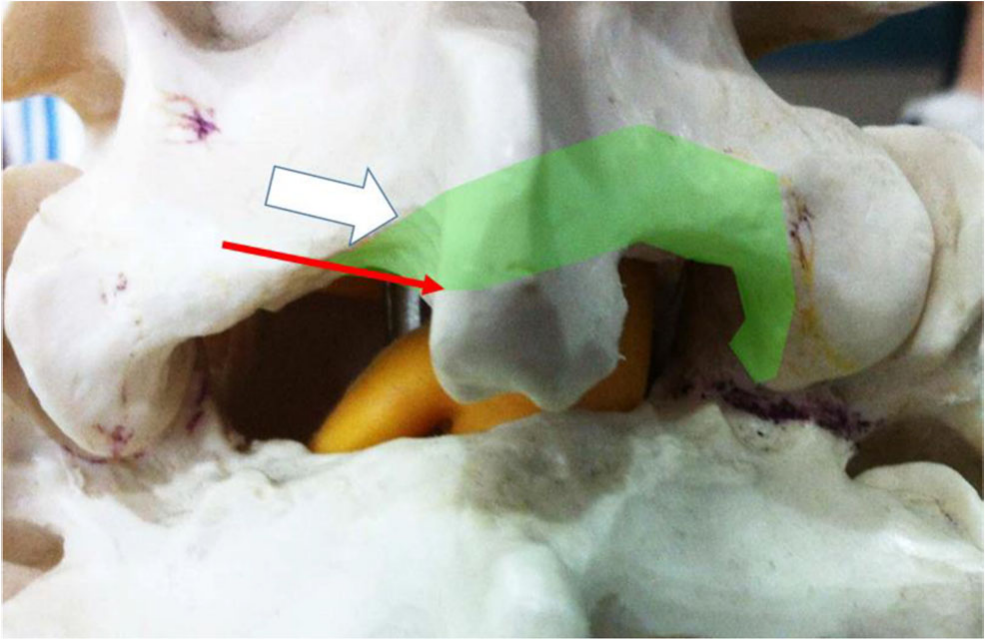
:Path of drilling by translaminar route (broad white arrow) and sublaminar route (narrow red arrow). Drilling is kept closed by “walking” on the lamina to avoid inadvertent neural compression. The path of drilling is as shown by the green region along the ventral lower half of the lamina to superior articular process and also the caudal lamina is drill till ligamentum flavum is detached. Ventral portion of interspinous ligament is also partly detached during drilling but will not affect stability of the spinal segment

With the sublaminar corridor, drilling focuses on the under surface of the spinolaminar junction. It is suitable for patients with minimal central and lateral recess stenosis, and it conserves most parts of the interspinous ligament and spinous process to provide stability. Another option is translaminar corridor which indicates if central and lateral recess compression involves drilling of the ventral base of the spinous process with partial detachment of the ventral portion of the interspinous ligament but is not preferred by the authors who preferred interspinous ligament conservation (Fig. 3).

### Use of intraoperative image guidance

Intraoperative imaging is used in checking for confirmation of crossing over to the contralateral side and adequacy of extraforaminal decompression (Fig. 2e, f).

### Systematic drilling in central and lateral recess (Figs. 4 and 5)

**Fig. 4:**
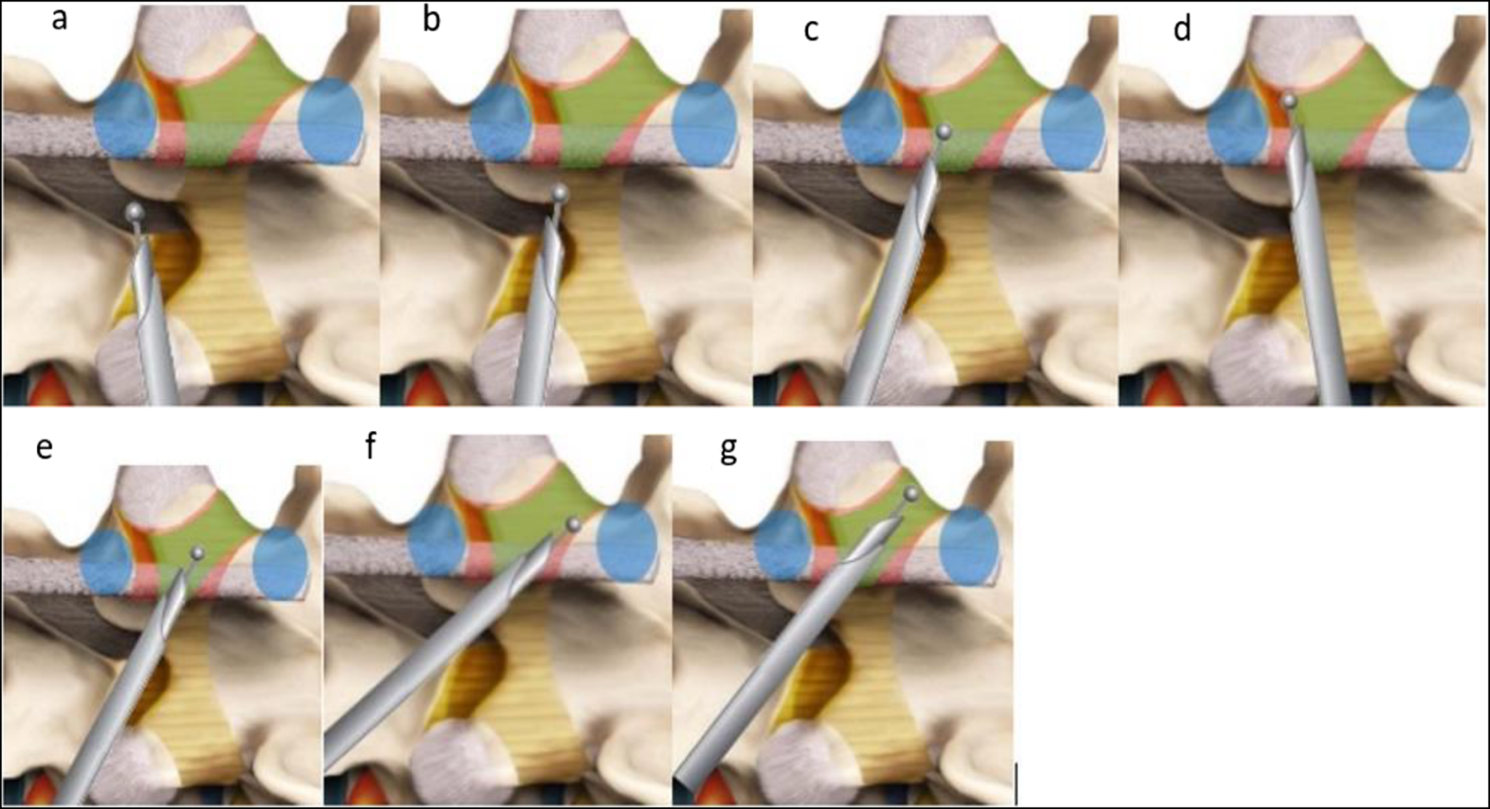
Sublaminar contralateral interlaminar foraminotomy. **a** Start drilling interspinous ligaments and creating a sublaminar space. **b** Drilling of the spinolaminar junction. **c** Drilling contralateral ventral lamina. **d** Drilling of superior articular process attachment of ligamentum flavum. **e**, **f** Drilling on proximal attachment of the ligamentum flavum. **g** Follow the ligamentum flavum out to the lateral attachment to allow full detachment of the ligamentum flavum. Blue signifies the pedicle, green signifies area of ligamentum flavum. Red is area of lamina

**Fig. 5:**
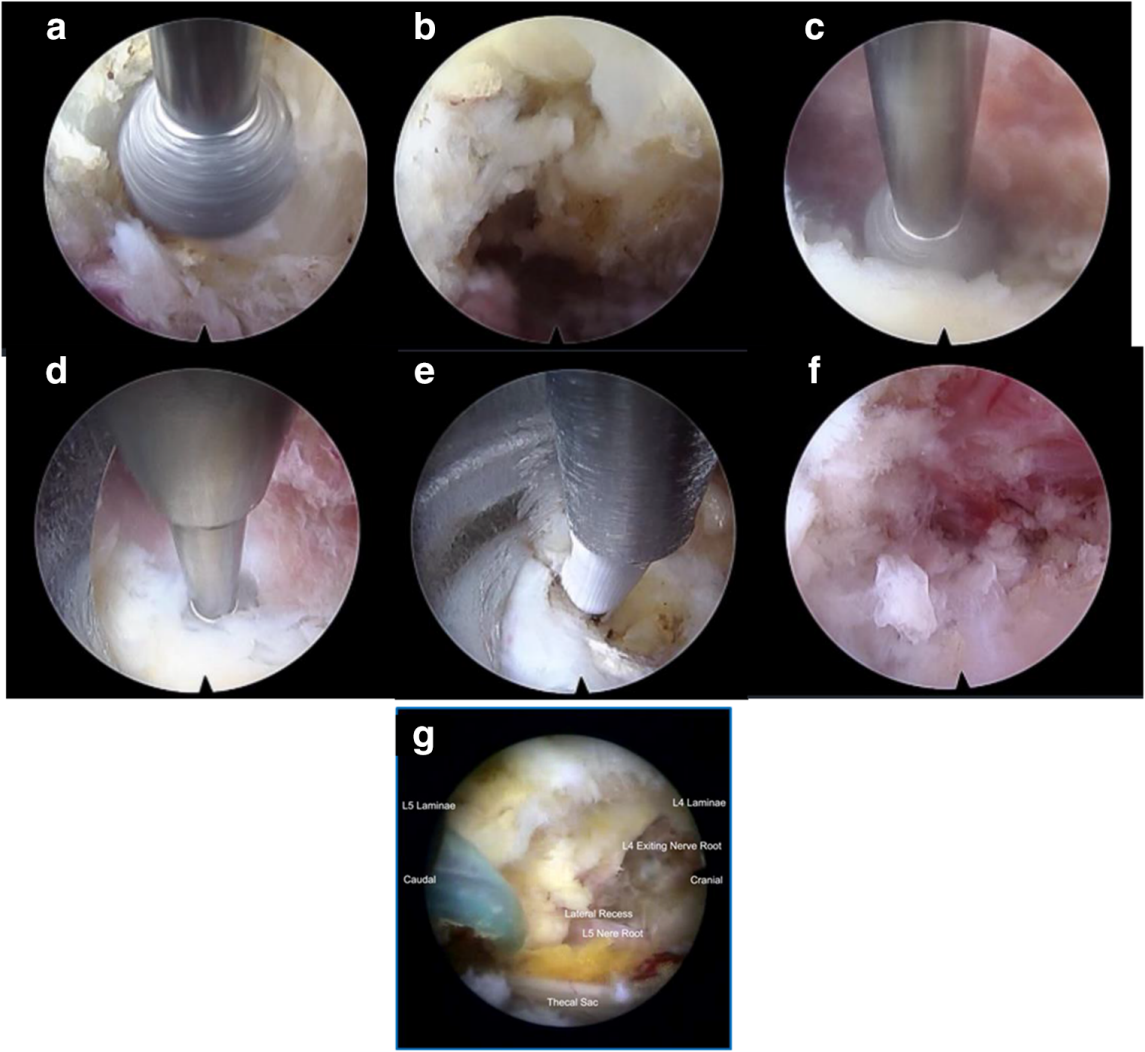
:**a** Docking and drilling of spinolaminar junction to create a sublaminar corridor. **b** Sublaminar corridor. **c** Contralateral ventral laminar drilling. **d** Drilling of contralateral superior articular process ligamentum flavum attachment. **e** Contralateral discectomy. **f** Trace the entire course of exiting nerve root ensuring it is well decompressed. **g** Clear contralateral decompression of L4/5 with L4 exiting nerve root and traversing L5 nerve root seen to be decompressed adequately

The sublaminar approach is described here. The systematic drilling technique is used with a 3.5-mm coarse diamond endoscopic burr on the base of the spinous process and ventral to the interspinous ligament at the spinolaminar junction to create a sublaminar space above the ligamentum flavum over the top of neural elements with the open bevel pointing upwards (Figs. 4a, b and 5a). Contralateral ligamentum flavum attachment is done on the cephalad and caudal lamina resected by drilling (Fig. 4c–f); the superficial layer of the ligamentum flavum was removed with forceps to have better visualization of the sublaminar space leaving a deep layer of the ligamentum flavum to protect neural elements till bony decompression is complete (Fig. 5b). Care must be taken to “walk” the drill close to the cephalad lamina dorsal to the flavum in the sublaminar space to avoid inadvertent compression of the underlying neural element (Fig. 5c). Resect lateral extension of the flavum was done at the ventral part of the superior articular process (Figs. 4d and 5d). Once a deep layer of the flavum is detached from all its attachment, it is removed with the endoscopic forcep.

### Foraminal and extra foraminal decompression (Figs. 4 and 5)

The exiting and traversing nerve root is seen after the flavum is removed. Any herniation of disk in the lateral recess is explored with radiofrequency and resected with endoscopic forceps (Fig. 5e). The foramen is explored with scope with drilling of the overriding superior articular facet dorsally, foraminal discectomy, and syndesmophyte resection (Fig. 4e). As the foramen is enlarged, the endoscope is introduced deeper into the foramen to explore any further compression and extra foraminal decompression if necessary (Fig. 4f, g) If necessary, the edge pedicles can be drilled with caution to free the nerve from entrapment. The authors termed this technique with no neural retraction necessary as “no touch” decompression of exiting nerve root.

### Final confirmation of completion of decompression

Complete decompression will have sign of pulsating nerve roots, the anemic nerve root turning pink, and return of natural angulation of exiting and traversing nerve roots (Fig. 5f, g).

**Fig. 6:**
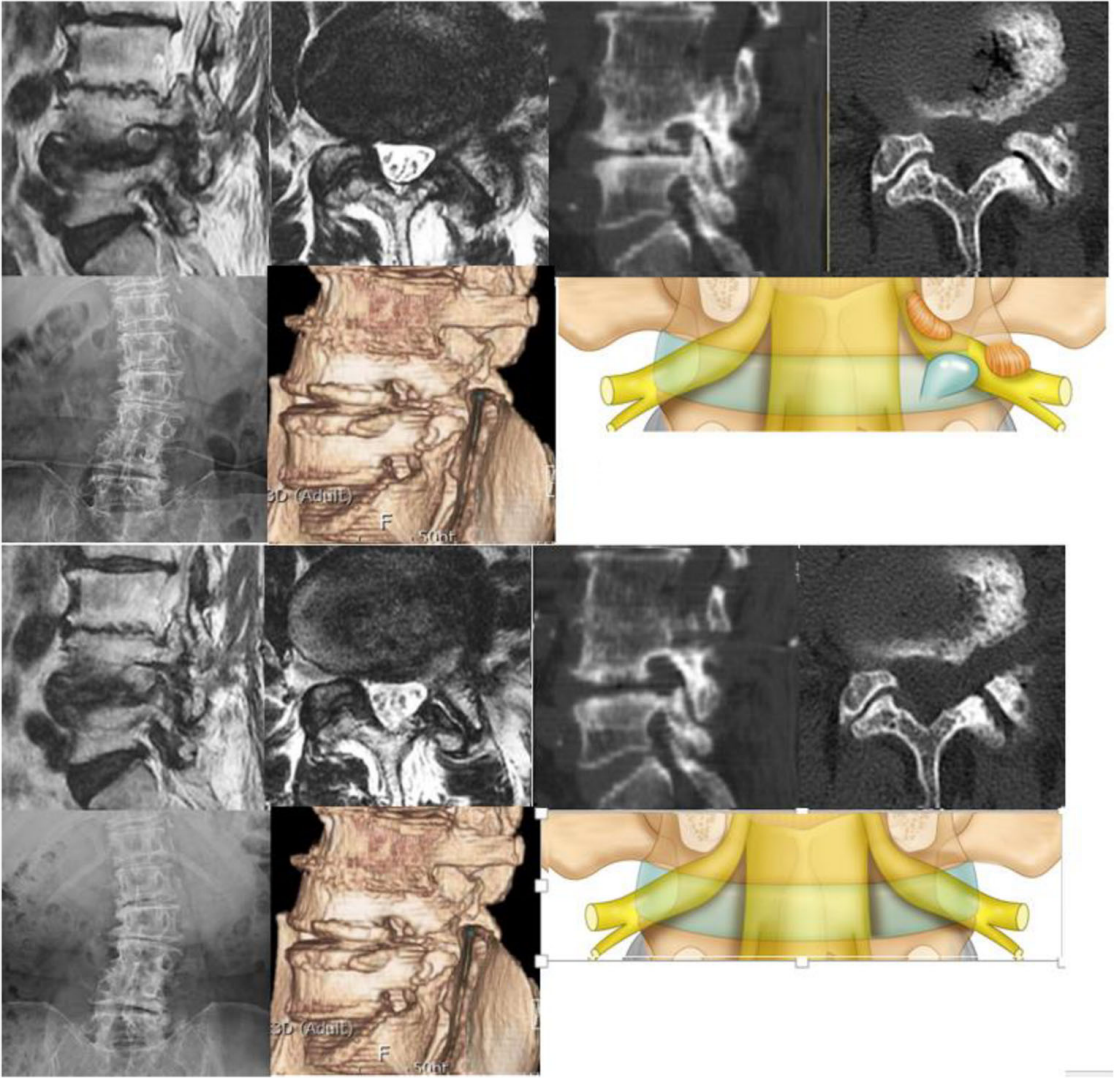
Case example of a 71-year-old female presented with left leg pain radiating to the dorsum of left foot. Top 7 pictures: radiological investigations showed a degenerative scoliosis with L4/5 on the left showing lateral wedging and concavity, CT scan showed overriding facet joint and decreased foraminal height as well as syndesmophyte impinging foramen and facet hypertrophy decreasing anteroposterior diameter of the foramen. MRI showing double crush especially in lateral recess, foramen and extra foramen. Illustration of the double crush sign is highlighted in pictoral form. Bottom 7 pictures showing adequate foraminal decompression in the axial and sagittal view. CT scan highlighting the effect of contralateral approach endoscopic decompression and XR showing no worsening of coronal deformity. Picture illustration of reversal of the double crush etiology


A case example shown (Fig. 6 and Video 1) showing contralateral L4/5 intervertebral foramen decompression.

ESM 1(MP4 92,649 kb)


### Indications

Symptomatic foraminal and/or extra foraminal stenosis. (Double Crush Syndrome).

### Limitations

Significant spinal instability with back dominant pain; fusion preferred. This procedure is relatively contraindicated in neurogenic tumor, infection and trauma related stenosis. Symmetrical lumbar spinal stenosis should consider bilateral decompression. [4]

#### How to avoid complications

1. Careful evaluation of CT and MRI scan deciding on how extensive is foramen exploration to prevent unnecessary destabilization of the spine segment concerned
2. Put the drill close to the ventral surface of the lamina and take care to avoid pressure on dura; if visualization is poor, consider removing the superficial layer of the flavum with punches and forceps keeping the deep layer protecting dura till flavectomy is complete.
3. Avoid thinning out the bone near the pars region above the attachment of the ligamentum which may need pars fracture
4. Avoid overzealous pedicle drilling to enlarge the foramen which may lead to pedicle fracture; if in doubt, do intraoperative roentgenogram [10].

### Specific perioperative considerations

Drain inserted prior to closure of skin to drain the saline fluid used for hydrostatic pressure irrigation during endoscopy as such fluid can potentially cause discomfort with soft tissue distension and potential chemical irritation. Drain is removed on post-operative day one.

Physiotherapy with the patient is mobilized on the next day as per the pain toleration.

### Specific information to give to the patient about surgery and potential risks

It is on the same level; the opposite side of the decompressed region stenosis is a possibility in the future. This is weighed against unnecessary decompression and disruption of ipsilateral unaffected side anatomy. Intimate working with dura can lead to dura tear, with a certain level of endoscopic experience required prior to attempt in this technique.

#### Summary of 10 key points

1. Advantages of endoscopic contralateral approach are “no touch” decompression of exiting nerve root, facet conservation, and ability to do foraminal and extraforaminal decompression from a single approach.
2. Double areas of spinal stenosis synergistically cause symptoms
3. Preoperative clinical and radiological assessment for gross anteroposterior and lateral instability
4. Lateral wedging and mild lateral listhesis are particularly suitable for the uniportal contralateral approach as an alternative to the paraspinal approach.
5. Oblique docking on the spinolaminar junction pointing towards the contralateral foramen
6. Try to apply the burr close to the ventral aspect of contralateral lamina to prevent pressure on underlying dura
7. Remove the deep layer of the flavum only after sufficient bony drilling
8. 30° endoscope for discectomy and foraminal and extra foraminal decompression
9. Check with an image intensifier for extent of extraforaminal decompression
10. End of decompression will visualize course of pulsating exiting nerve out to extraforaminal region.
